# The Effects of the Amount of Information on Episodic Memory Binding

**DOI:** 10.5709/acp-0188-z

**Published:** 2016-06-30

**Authors:** Frine Torres-Trejo, Selene Cansino

**Affiliations:** Laboratory of NeuroCognition, Faculty of Psychology, National Autonomous University of Mexico, Mexico City, Mexico

**Keywords:** binding, episodic memory, inter-item, associative recognition, cued-recall

## Abstract

The effects of increasing the number of items to be remembered on associative recognition and cued recall were examined. Thirty participants were asked during encoding to determine whether two- and three-item stimuli contained natural objects, artificial objects, or both. In an associative recognition task, the participants indicated whether the stimuli were identical to those presented during encoding, were rearranged by exchanging one of the two-item stimuli for one of the three-item stimuli, or represented a new stimulus. The correctly identified rearranged item pairs and triads were included in a subsequent cued-recall task in which participants verbally reported the missing item. As the number of items increased, the discrimination of rearranged stimuli diminished, but that of identical trials remained the same. Furthermore, the ability to retrieve the missing item was unaffected. It was concluded that the effect of the amount of information on binding depends on how the information must be retrieved.

## Introduction

 The ability to remember multiple events from past experience, as well as the contexts in which they took place, such as the time or location, is one of the most important attributes of episodic memory. The integration of several elements into complex memory representation is accomplished through a binding process (Mather, 2007). According to relational memory theory (Cohen & Eichenbaum, 1993), multiple elements from an episodic experience may be integrated in a flexible binding in which each element preserves its individuality because it is accessible at retrieval, along with the relationship between the elements. Conversely, a binding may be a unitary representation in which the elements and their relationships are unitized and thus retrieved as a whole (Sutherland & Rudy, 1989). The ability to retrieve events and their context from a flexible binding relies on recollection, whereas memories that lack these details are based on familiarity. The identification of a unitized binding by means of familiarity occurs only for the intrinsic features of an event ( Yonelinas, Kroll, Dobbins, & Soltani, 1999). 

 During encoding and subsequent retrieval, the binding process differs depending on the complexity of the episodic event. The most essential binding process is based on the intrinsic (intra-item) features of a single item within an episodic event and occurs automatically and effortlessly when the item is perceived (e.g., Hommel, 2005; Hommel, Müsseler, Aschersleben, & Prinz, 2001; Treisman, 1996; Troyer & Craik, 2000; Zimmer & Ecker, 2010). In contrast, the formation of complex episodic representations involving several items and contexts requires active attentional and monitoring processes (e.g., Moeller & Frings, 2014a, 2014b; Uncapher, Otten, & Rugg, 2006; Zimmer & Ecker, 2010). 

 Item-context binding for one (e.g., Düzel, Yonelinas, Mangun, Heinze, & Tulving, 1997; Gutchess et al., 2007; Tsivilis, Otten, & Rugg, 2003; Van Petten, Senkfor, & Newberg, 2000) or two (e.g., Estrada-Manilla & Cansino, 2012) contexts has been studied extensively. Inter-item bindings—that is, the binding of two independent objects that may belong to the same domain (e.g., two faces) or to different domains (e.g., house and apple) (Mayes, Montaldi, & Migo, 2007) are more difficult to remember than item-context bindings (Piekema, Rijpkema, Fernández, & Kessels, 2010). The studies using words (e.g., Curran, 2000; Park & Rugg, 2008; Rhodes & Donaldson, 2007) have provided clear evidence that binding benefits from a semantic relationship between the stimuli (e.g., Curran, 2000; Rhodes & Donaldson, 2007). In the current study, we employed perceptually rich color images to study the binding of visual information. 

 The most classic procedure used to study inter-item binding is the associative recognition task, which consists of presenting pairs of items that are presented identically or recombined with an item from another pair in the test. Because all items have been previously presented, recombined or not, familiarity does not provide a guide for recognizing these two types of inter-item bindings; therefore, recollection is needed to perform the task (Mecklinger & Jäger, 2009). Rearranged pairs are usually more difficult to recognize than intact pairs (e.g., Badgaiyan, Schacter, & Alpert, 2002; Greve, van Rossum, & Donaldson, 2007). Several interpretations have been put forward to explain the advantage of intact pairs. One proposed interpretation is that intact pairs benefit from the match between the stimulus pair and the original encoding information (Clark & Shiffrin, 1992). Their advantage also has been attributed to the associative information between items. This point of view assumes that once one of the items is recognized, the association of that item with the other facilitates its recognition in intact, but not rearranged stimuli (Humphreys, 1976). 

 The research on episodic memory binding has focused mainly on successful recollection; however, recollection often fails. Binding failures have been identified by several authors (e.g., Roediger & DeSoto, 2001; Schacter, Norman, & Koutstaal, 1998), but few studies have attempted to characterize these failures or determine their rate of occurrence (Reinitz & Hannigan, 2004; Reinitz, Lammers, & Cochran, 1992). Binding errors have been extensively studied using the memory conjunction error paradigm (Underwood & Zimmerman, 1973), mainly with verbal material (Jones & Atchley, 2006), but sometimes with other types of stimuli, such as faces (Jones, Bartlett, & Wade, 2006) or abstract figures (Kroll, Knight, Metcalfe, Wolf, & Tulving, 1996). The task consists of presenting compound words to study; then, at testing, single words derived from the compound words are mixed with other words. Because all single words were previously presented, the participant perceives that the rearranged compound word was presented earlier (conjunction errors). This procedure permits the study of intra-item binding errors because, at encoding, the compound words are unitized stimuli that represent a single meaning. 

 According to some authors (Jones & Jacoby, 2005; Jones, Jacoby, & Gellis, 2001), conjunction errors arise because only part of the memory trace for a particular stimulus is remembered, and this memory failure contributes to incorrect combinations of previous stimuli (Schooler & Tanaka, 1991). The associative recognition task may be used to study inter-item binding errors—that is, errors induced by combining items from different stimulus pairs. However, errors in this task provide little information about the mechanisms that may fail during the binding process. Conversely, the examination of the varied errors generated in free- or cued-recall tasks allows the disentanglement of the possible reasons for binding failure. 

**Figure 1.**
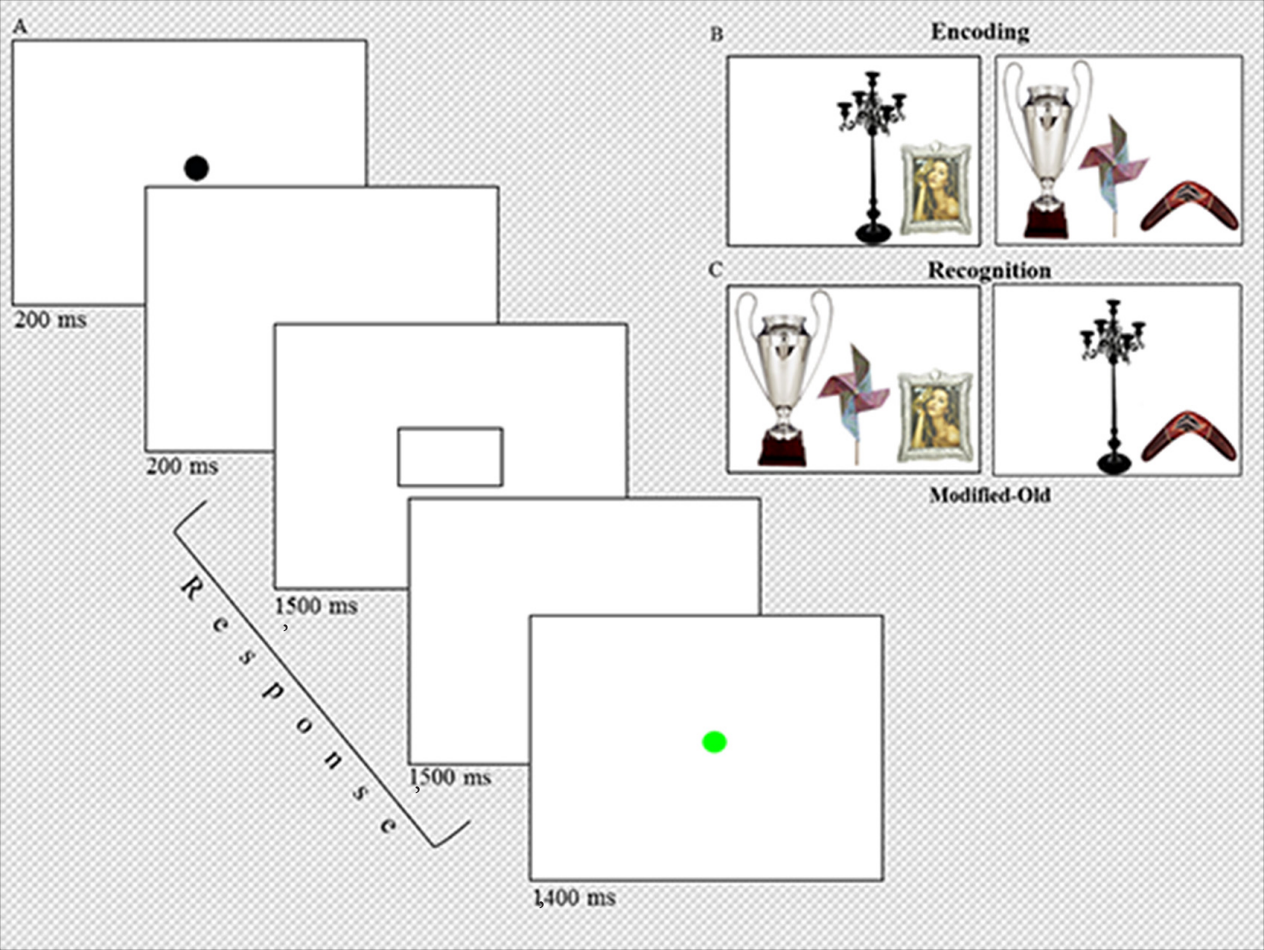
Events during each trial at encoding and recognition (A). Examples of the two- and three-item stimuli used at the encoding phase (B). Examples of rearranged-old stimuli, in which one of the items from the two-item stimulus has been exchanged for one of the items of the three-item stimulus in the recognition phase (C).

 Remarkably, none of the previous inter-item studies have directly contrasted the effects of increasing the number of items on associative recognition. Two previous studies (Clark, 1992; Clark & Shiffrin, 1992) included pairs and triplets of words. However, memory performance was not directly contrasted between the two types of stimuli. The aim of the present study was to examine the effects of the number of items on the binding of episodic memory representations. To this end, two and three images of common objects were randomly presented during encoding, and the participants determined whether the two or three images represented natural, artificial or both natural and artificial objects (Figure 1). Subsequently, one of the images from the pairs in half of the stimuli was replaced with one of the images from the triads. Stimuli comprising new items were also included. The participants indicated whether each pair or triad was intact, rearranged, or new compared with the stimulus presented at encoding. The rearranged stimuli that were correctly identified were presented again in a subsequent cued-recall task in which participants were required to verbally report the image missing in each pair and triad. 

The second aim of this study was to characterize the type of errors that were generated when we attempted to reconstruct the original information presented and how these errors were affected by the number of items. To achieve this goal, the different errors produced during the cued-recall task, as well as the correct responses, were examined. In particular, we distinguished among the correct retrieval of the missing original item (correct), retrieval of another item from the same pair or triad to which the exchanged item originally belonged in the study phase (within-error), retrieval of items from other pairs or triads (between-error), and no-remember responses (no-remember).

The precision of task performance decreases and reaction time increases in associative recognition tasks when the original inter-item presentation is rearranged. However, whether the ability to recall three items is equivalent to that for two items when the stimulus is either intact or rearranged remains an open question. To the best of our knowledge, the direct contrast of binding two and three items in the same study has not yet been examined. Moreover, it is also unknown whether recollection in cued-recall tasks is affected when the number of items increases, and if so, whether the incidence of the different errors varies as a function of the amount of information.

 Consistent with previous inter-item studies (Speer & Curran, 2007), we expected that participants’ discrimination levels (*d*’) would decrease and that reaction times would increase in the associative recognition task for rearranged items compared with intact items for both two- and three-item stimuli. In addition, we expected that the discrimination of rearranged and intact stimuli would be higher for the two-item compared with the three-item stimuli. This is because increasing the number of items may affect the binding process, as each item requires scanning and comparison with the other items constituting the memory representation. 

Because no previous study has examined two- and three-item stimuli in a cued-recall task, we have no a priori prediction of whether the increase in the amount of information will affect the retrieval of the missing item. We expected that retrieving the missing item in the cued-recall task would be more difficult for three-item than two-item stimuli because as the number of items increases, an individual must store the information related to an additional item as well as its relationship with the other items. Therefore, the increase in the amount of information will produce more interference and consequently affect the retrieval of the relevant item.

## Experiment

### Method

#### Participants

Thirty adults (15 women) with an *M*_age_ = 24.8, *SD* = 2.6 years and *M*_age_ = 16.5, *SD* = 1.7 years of formal education participated in this study. All participants reported no diagnosis of psychiatric or neurological disorders, no drug addiction, and no consumption of drugs that alter central nervous system functions during the previous six months. The participants had normal or corrected-to-normal visual acuity. The research protocol was approved through the Bioethics Committee of the Faculty of Medicine at National Autonomous University of Mexico. All participants provided written informed consent.

#### Stimuli

A total of 900 color images of common objects were used to build 360 stimuli: 180 stimuli containing two images (two-item stimuli) and 180 stimuli containing three images (three-item stimuli). The two- and three-item stimuli were examined by 50 individuals with the same characteristics as the participants in the current study to ensure the images used in the study were easy to identify and nameable. Additionally, to avoid providing clues to aid the remembrance of the images, each pair and triad included images that were of a proportional size (e.g., dog/chair) and could be encountered in everyday life (e.g., rose/book) but did not have an obvious relationship (e.g., computer/mouse). Thus, none of the stimuli had images with an odd relationship (e.g., horse/bed) or different size proportions (e.g., pencil/elephant). All stimuli, pairs and triads were presented within a black frame with 5.15° × 2.0° horizontal and vertical visual angles, respectively. The images with three-item stimuli filled all three positions inside the frame, whereas two-item stimuli were located in two of the three possible positions. The three possible locations for the two-item stimuli (i.e., left-middle, left-right, and middle-right) were used with the same probability (Figure 1). For both two- and three-item stimuli, 60 stimuli contained images representing natural objects, 60 stimuli contained images representing artificial objects, and the remaining stimuli contained images representing both natural and artificial objects. In the encoding phase, 120 two- and 120 three-item stimuli were presented, and in the recognition phase, 180 two- and 180 three-item stimuli were presented. In the recognition phase, for two- and three-item stimuli, 60 images were intact (i.e., equal to those presented in the encoding phase), 60 images were rearranged by exchanging one of the images in the two-item stimuli with one of the images in the three-item stimuli, and 60 images were new (i.e., not seen previously in the experiment). Images representing natural objects were always exchanged with other images representing natural objects; the same procedure was used for images representing artificial objects. The stimuli were presented in 12 blocks comprising an encoding (20 stimuli) phase and a recognition (30 stimuli) phase. The same number of all types of stimuli was included in each block from both phases, and all types of stimuli were presented in random order.

#### Procedure

The memory task was performed in a sound-dampened room. Two box panels were used, and each panel was placed on one of the armrests of a high-back chair. One of the panels had two push buttons, and the other panel had only one push button. The same events and exposure time were used in the encoding and recognition phases. Each trial began with a black circle as a fixation point (200 ms), followed by a blank screen (200 ms). The stimuli were then displayed at the center of the screen (1,500 ms), followed by a blank screen (1,500 ms). A green circle was presented during a rest period between trials (1,400 ms). The participants responded from the onset of the stimuli and during the following 3,000 ms. During encoding, the participants were asked to indicate whether all of the images in the two- or three-item stimuli were natural, artificial, or a combination. During the recognition phase, the task was to indicate whether the stimuli were intact, rearranged, or new. In each phase, the three possible answers were independently counterbalanced between the middle and index finger of the left or right hand and the index finger of the other hand. Therefore, for all participants, the key assigned for one type of response in encoding did not correspond to the same type of response at recognition. At the beginning of the study, the participants performed brief versions of both phases as practice.

After each block, the stimuli correctly identified as rearranged were presented again in a self-paced retrieval phase in which the participants were asked to verbally describe the exchanged and missing images in both the two- and three-item rearranged stimuli.

#### Data analysis

Recognition hits for two- and three-item stimuli, regardless of the type of trial (intact or rearranged), were submitted to paired Student’s *t*-tests. The same analysis was conducted for new items that were classified as old items. The data from the recognition task were analyzed using *d*’ values because they provide an accurate estimate of the participants’ ability to discriminate between signal and noise. Before computing *d*’ values, to control for hit recognition differences between pairs and triads, the intact accuracy and rearranged accuracy for two-item stimuli were estimated as the percent of correct recognition hits for pairs that received a correct intact or rearranged response, respectively. The same procedure was used to estimate these responses for three-item stimuli. The false alarm rate used to calculate *d*’ for the intact two- and three-item stimuli was the probability of responding “rearranged” to an intact stimulus, whereas the probability of responding “intact” to a rearranged stimulus was the false alarm rate used to estimate *d*’ for rearranged stimuli. Likewise, *c * values were estimated to measure the response bias. The *d*’ and *c * values were examined separately through repeated-measures analyses of variances (ANOVAs), which included the number of items (two or three) and the trial type (intact or rearranged) as factors. A similar procedure was used to analyze the reaction times for hits in the associative recognition task. Likewise, a repeated-measures ANOVA was conducted for data from the cued-recall task using the number of items (correct recognition of rearranged two- and three-item stimuli) and response type (correct: retrieval of the original item, within-error: retrieval of another item from the same pair or triad to which the item exchanged originally belonged in the study phase, between-error: retrieval of items from other pairs or triads, and no-remember) as factors. The performance in the encoding phase was analyzed using Student’s *t*-test. When necessary, the degrees of freedom were corrected using the Greenhouse-Geisser procedure. In these cases, the coefficient ε, original degrees of freedom, and corrected probability levels are reported. Post hoc comparisons were performed using Scheffe’s test to elucidate significant differences between the three level factors and interactions. The significance level was *p* < .05.

### Results

#### Encoding

The mean percent of correct responses was equivalent, *t*(29) = 1.68, *p* = .10, for two-item (*M* = 89.0, *SE* = 0.31) and three-item (*M* = 88.0, *SE* = 0.33) stimuli. The reaction times (RTs) significantly differed, *t*(29) = -8.88, *p* < .0001: the responses for three items (*M* = 1,427, *SE* = 33) took longer than that for two items (*M* = 1,314, *SE* = 29).

#### Recognition

Recognition hits differed significantly, *t*(29) = 8.38 *p* < .001, for two- (*M* = 73.59, *SE* = 1.69) and three-item (*M* = 66.92, *SE* = 1.50) stimuli; however, incorrect responses for new items did not differ, *t*(29) = -0.86, *p* = .39; for two-item: *M* = 12.20, *SE* = 2.05, three-item: *M* = 13.28, *SE* = 2.15.

 The repeated-measures ANOVA conducted on *d*’ values was significant for the factor trial type, *F*(1, 29) = 6.84, *MSE * = 0.18, *p* = .01, η_p_^2^ = .19, and for the interaction between trial type and number of items, *F*(1, 29) = 5.30, *MSE * = 0.02, *p* = .03, η_p_^2^ = .16, but not for number of items, *F*(1, 29) = 0.91, *MSE * = 0.18, *p* = .35, η_p_^2^ = .03, 95% CI = 1.12 <= µ1 - µ2 <= 1.46. The *d*’ values were higher for intact (1.40 ± 0.11) than for rearranged (1.20 ± 0.09) trials. Post hoc Scheffe’s tests showed that *d*’ values were higher for intact trials compared to rearranged trials for both two- and three-item stimuli (Table 1). Likewise, *d*’ values for rearranged trials were higher for two-item stimuli than for three-item ones; however, intact trials did not differ between pairs and triads. To further confirm that the null effect of set size on the intact trials was supported by the data, we conducted a Bayesian analysis (Masson, 2011). First, we carried out an ANOVA on *d*’ values for the intact two- and three-item stimuli to obtain the appropriate sum-of-squares values. The resulting Bayes factor was 5.42 and the posterior probabilities were P_BIC(HO|D)_ = .84 and P_BIC(H1|D)_ = .16 for the null and alternative hypotheses, respectively. According to Raftery’s (1995) classification, this outcome provides positive evidence in support of the null hypothesis. The analysis conducted on *c * values was significant for trial type, *F*(1, 29) = 6.84, *MSE * = 0.04, *p* = .01, η_p_^2^ = .19, and for the interaction between trial type and number of items, *F*(1, 29) = 5.30, *MSE * = 0.006, *p* = .03, η_p_^2^ = .16, but not for number of items, *F*(1, 29) = 0.91, *MSE * = 0.18, *p* = .35, η_p_^2^ = .03. The main effect of trial type revealed that *c * values were higher for rearranged (.70 ± .05) than for intact (.60 ± .05) trials. The post hoc tests computed to examine the significant interaction showed that the *c * values were higher for rearranged trials than for intact ones for both two- and three-item stimuli (Table 1). Additionally, *c* values for intact trials were lower for pairs than for triads, but rearranged trials did not differ. 

**Table 1.** Participant Performance in the Associative Recognition Task

	Hits	False alarm	Incorrect new	*d*’	*c*
Two-item					
Intact	77.50 (12.76)	20.50 (1.49)	2.07 (1.40)	1.40 (.12)	.63 (.05)
Rearranged	69.67 (12.83)	25.23 (2.45)	5.12 (0.75)	1.27 (.10)	.70 (.06)
Three-item					
Intact	73.00 (12.73)	23.50 (0.81)	3.60 (0.50)	1.39 (.11)	.56 (.05)
Rearranged	60.85 (16.97)	30.81 (2.44)	8.41 (2.19)	1.13 (.11)	.70 (.06)

*Note*. False alarm rates correspond to rearranged responses for intact stimuli and intact responses for rearranged stimuli. Incorrect new responses are intact and rearranged stimuli that were judged as new. Standard deviations are shown in parentheses.

The analysis conducted on the RTs for correct responses was significant for number of items. *F*(1, 29) = 5.65, *MSE * = 23,155.98, *p* = .02, η_p_^2^ = .16, but neither for trial type, *F*(1, 29) = 3.58, *MSE* = 52,594.20, *p* < .07, η_p_^2^ = .11, nor for the interaction between the two factors, *F*(1, 29) = 0.48, *MSE * = 26,306.57, *p* < .49, η_p_^2^ = .02. The participants were faster on the two-item stimuli(mean ± *SE*: 1,439 ± 25) than on the three-item stimuli (1,505 ± 36).

#### Cued recall

**Figure 2.**
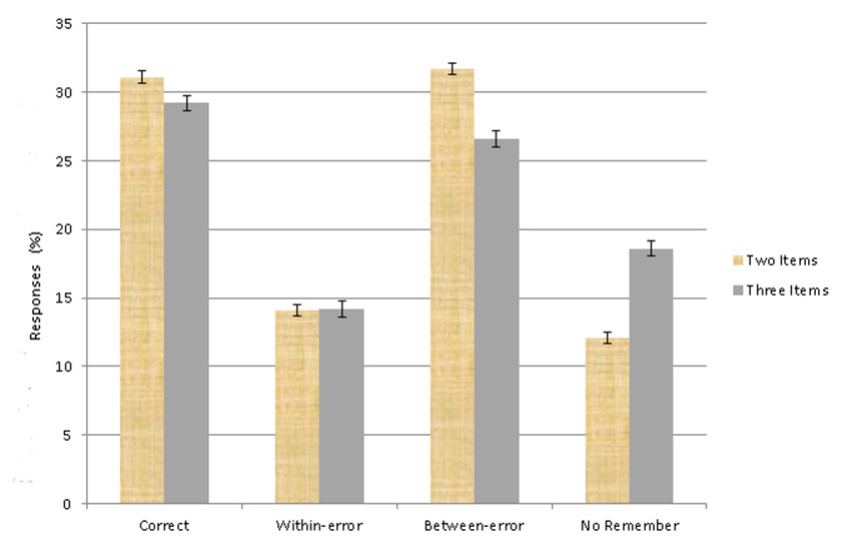
Performance in the cued-recall task; the two- and three-item stimuli only differed significantly in between-error rates and no-remember response rates. Significant differences between response types are described in the text. Error bars represent standard errors.

Only the correctly identified old-rearranged stimuli were used in the cued-recall task: The mean number of two-item stimuli was 41.8, and the mean number of three-item stimuli was 36.5. The ANOVA results were significant for response type, *F*(3, 87) = 775.79, *MSE* = 6.58, ε = .87, *p* < .001, ε = .87, η_p_^2^ = .96, and for the interaction between response type and the number of items, *F*(3, 87) = 53.34, *MSE * = 8.88, *p* < .001, ε = .77, η_p_^2^ = .65, but not for number of items, *F*(1, 29) = 0.13, *MSE * = 4.14, *p* = .72, ηp2 < .004, 95% CI = 21.8 <= µ1 - µ2 <= 22.7. Post hoc Scheffe’s test analyses revealed that for the main effect of the factor response type, correct responses (30.17 ± 0.25) significantly differed from within-errors (14.14 ± 0.37) and no-remember responses (15.38 ± 0.19), within-errors differed from between-errors (29.15 ± 0.44), and between-errors differed from no-remember responses. The post hoc analysis conducted to elucidate the significant interaction showed that between-error rates were higher for two-item stimuli than for three-item stimuli, whereas no-remember response rates were higher for three-item stimuli than for two-item ones (Figure 2). Post hoc analyses also showed that for two-item stimuli, correct response rates were higher than all types of errors, except for the between-errors, whereas for three-item stimuli, correct response rates were superior to all types of errors. The between-error rates were higher than all types of error rates for both two- and three-item stimuli. Within-error rates were higher than no-remember response rates for three-item stimuli, but not for two-item stimuli. Because the correct response rates were unaffected by the number of items, we conducted a Bayesian analysis to estimate the degree of evidence supporting the null hypothesis. An ANOVA for the correct two- and three-item stimuli was calculated to obtain the correct sum-of-squares values. The Bayes factor was 3.13, and the corresponding posterior probabilities for the null and alternative hypotheses were P_BIC(HO|D)_ = .76 and P_BIC(H1|D)_ = .24, respectively. This outcome provides positive evidence in support of the null hypothesis. Three other types of errors were identified, but they were not analyzed because of their low incidence: the incorrect identification of the item that was replaced and retrieval of an item from a different pair or triad (two-item: 3.1 ± 0.18, three-item: 3.3 ± 0.15), the correct retrieval of the original bound item but incorrect identification of the item that was replaced (two-item: 3.6 ± 0.15, three-item: 3.4 ± 0.20), and the retrieval of an item presented in a new pair or triad (two-item: 3.1 ± 0.20, three-item: 3.2 ± 0.12). Additionally, participants denied that 1.2% of two-item and 1.5% of three-item stimuli were rearranged.

**Table 2.** Reaction Times in the Associative Recognition Task

	Hits	False alarm	Incorrect new
Two-item			
Intact	1,389 (155)	1,377 (161)	1,303 (198)
Rearranged	1,489 (190)	1,706 (160)	1,403 (196)
Three-item			
Intact	1,475 (177)	1,595 (226)	1,693 (180)
Rearranged	1,534 (318)	1,679 (164)	1,713 (199)

*Note*. False alarm rates correspond to rearranged responses for intact stimuli and intact responses for rearranged stimuli. Incorrect new responses are intact and rearranged stimuli that were judged as new. Standard deviations are shown in parentheses.

### Discussion

The most relevant findings of this experiment were that an increase of information decreased the participants’ performance in the associative recognition task for rearranged, but not intact, stimuli. Moreover, increasing the number of items had no effect on the participants’ abilities to retrieve the correct missing item in the cued-recall task. However, certain types of errors, particularly between-errors and no-remember responses, were sensitive to the amount of information.

 As predicted, the ability to discriminate rearranged trials diminished for triads relative to pairs; however, opposite to our expectations, the discrimination of intact trials was unaffected by the number of items. The fact that the amount of information had different effects on intact and rearranged trials cannot be attributed to the utilization of different processes, such as familiarity and recollection, to identify each of these types of trials because performance in associative recognition tasks mainly relies on recollection, given that all studied items are familiar (Mecklinger & Jäger, 2009; Yonelinas, Aly, Wang, & Koen, 2010). Therefore, familiarity may be an inefficient process to discriminate between intact and rearranged trials. Moreover, there is little possibility that participants treated the items of pairs and triads as a single unitized item to solve the task using familiarity because the items within each stimulus were highly unrelated, and unitizing requires encoding of the items into a single conceptual unit (Yonelinas et al., 2010). 

 The lack of size-effects on intact trials may be explained by the proposal that each item from an associative trial is encoded individually, along with the association between the items, into an episodic representation (Diana, Reder, Arndt, & Park, 2006). At recognition, each item from an intact trial triggers the same episode; while in rearranged trials, each item activates its own episode. Therefore, rearranged trials are more difficult to recognize because the individual items accumulate less information from the original episode than intact trials. According to this proposal, intact two- and three-item stimuli are retrieved with the same efficiency because all items within stimuli activate the same original episode. Conversely, rearranged trials were affected by set size because items within each stimulus initiate the retrieval of different episodes, and three-item stimuli collect less information from the original episode than two-item stimuli. As in previous studies (Clark, 1992; Humphreys, 1976), intact trials were easier to identify than rearranged trials. The current study not only confirms the benefit of intact trials over rearranged ones but also provides further evidence that this advantage is preserved, even if the amount of information to be retrieved increases. 

Moreover, the small effect size (η_p_^2^ < .03) and minor difference between 95% confidence intervals (*d* = .34) suggest that discrimination between intact two- and three-item stimuli is relatively insignificant. Likewise, the Bayesian model selection analysis revealed that the data provide evidence in favor of the null effect of the set size on the intact trials.

 The theory of event coding (Hommel, 2005 ) proposed that not only stimulus representations but also the responses associated into a memory representation can be automatically retrieved if some information of that representation is again experienced (Hommel et al., 2001). However, the benefit of encountering the same information did not shorten the RT. Participants took the same time to identify intact and rearranged trials, indicating that this advantage does not act as an automatic or effortless process. Moreover, three-item stimuli were more time consuming to identify than two-item stimuli for both types of trials, as revealed by the longer RTs for the former. This outcome suggests that participants actually scanned each item within the stimulus. 

 It is possible that the task employed during the encoding phase weakened the binding process because we asked the participants to judge whether the items were natural, artificial, or both. Thus, this encouraged the processing of each item independently, and there is evidence that the association between items is reduced when they are processed individually (Henke, Buck, Weber, & Wieser, 1997). This strategy was adopted to ensure that all items within the stimulus were equally attended and perceived. Moreover, this procedure allowed us to maintain the same encoding task across stimuli and participants, which provides control that is difficult to achieve when participants are requested to freely associate stimuli for a subsequent memory task. 

 In the current study, rearranged stimuli were built by exchanging one item from a pair with one item from a triad. Because the stimuli were originally presented with a different number of items, the question of whether this procedure influenced the higher discrimination level observed for intact trials relative to rearranged trials has been raised. This outcome may not be attributed to the exchange of items between different set size stimuli because our results are consistent with previous studies that have used an associative recognition task consisting of pairs (Badgaiyan et al., 2002; Clark, 1992; Greve et al., 2007; Humphreys, 1976). Moreover, for both two- and three-item stimuli, only one item was exchanged; thus, the exchange was maintained constant across set-sizes. 

 Recollection in the cued-recall task demonstrated that a single item can be retrieved independently of the number of items bound. This outcome indicates that increasing the amount of information by adding one item and its relationship with the other items did not increase interference to a degree that would disrupt the retrieval of the missing item. Likewise, this finding suggests that individuals were able to encode the items in a flexible binding memory representation that allowed them to independently retrieve the missing item ( Moses & Ryan, 2006) and that these flexible bindings were unaffected by the complexity of the stimuli. However, the proportion of correctly reported missing items was approximately 30% for both the two- and three-item stimuli, which suggests that the ability to accurately retrieve the original episodic experience is a highly demanding task that predisposes participants to produce incorrect responses. 

The effect of the amount of information on the participants’ performance for rearranged trials in associative recognition, but not in cued recall, may be attributed to the employment of different strategies during each task because of the RT. In the cued-recall task, participants had an unlimited amount of time to retrieve the missing item, and therefore all resources and strategies may have been involved in solving the task. In contrast, in the associative recognition task, participants were under time pressure to respond, and this situation might have induced more misses and false alarms, as defined in the current study.

 Moreover, the fact that retrieving the missing item was unaffected by the size of the binding indicates that the other items from the stimuli did not function as cues to enhance the retrieval of the missing items. In item-context studies, it has also been observed that retrieving one context does not necessarily facilitate the retrieval of a second context (Starns & Hicks, 2005). The lack of a size effect on the cued-recall task can be explained by the fact that the task consisted of retrieving only one item from the bound set; therefore, the number of items included was irrelevant. This outcome is based on a difference between 95% confidence intervals of less than 1% for correct responses for two- and three-item stimuli. Furthermore, according to the Bayesian analysis, the lack of an effect of set size on the rates of correct responses was supported by the data, which provides support for the null hypothesis and not the alternative hypothesis. However, null results must be interpreted with caution, and further research is needed to confirm this finding. 

 Although the amount of information for cued-recall had no effect on the proportion of correct responses, effects were observed for between-errors and no-remember responses. In particular, between-error rates were higher for two- than for three-item stimuli, whereas no-remember response rates were higher for three- than for two-item stimuli. Within-error rates indicate that the items from the original pair or triad were encoded, but the association between the items was lost because individuals were unable to retrieve the relevant missing item from the original stimulus. Thus, in within-errors, only part of the original experience was lost. Conversely, between-errors are based on items that participants had observed across the task but that did not belong to the original pair or triad. Therefore, the studied episode was forgotten in this type of error. Although less original information is forgotten in within-errors compared to between-errors, both types of errors may be conceived of as misattributions (Jacoby, Kelley, & Dywan, 1989; Roediger, 1996; Schacter, 1999)—that is, an inability to retrieve the original experience leading to wrong memory reconstructions. In contrast, no-remember responses may be conceived of as truly forgotten or as a result of retrieval blocking (Roediger & Neely, 1982; Schacter, 1999) and thus constitute a complete memory fail. 

 In the current study, no-remember responses were more frequent for triads than for pairs, indicating that there was a higher probability of forgetting the missing item as the item number increased. In contrast, participants were more likely to engender between-errors for pairs than for triads, suggesting that when fewer items were encoded, participants attempted to retrieve the missing item. The between- and within-errors show how memories are likely to be reconstructed in quotidian situations when individuals directly attend to the information because the encoding task used in the current study requires all items to be fully attended, an important requirement of binding memory (Boywitt & Meiser, 2012; Reinitz, Morrissey, & Demb, 1994). Surprisingly, participants were more inclined to falsely reconstruct the episodic representations than to admit that they had forgotten the item that was missing. 

In conclusion, the recollection of an episodic memory representation is facilitated when its subsequent encounter is presented as it was originally encoded, independent of its complexity; however, when it is presented differently, the reconstruction of the original episode is more demanding and sensitive to the information contained in the original memory representation. These results also provide evidence that only some of the information we jointly experience and encode into our episodic memory representations is accurately retrieved. A certain proportion of our memories are false reconstructions of our previous experiences, and the remaining episodes simply disappear from our memory.
